# Ceftriaxone-induced symptomatic cholelithiasis in a child: case report and literature review

**DOI:** 10.1093/omcr/omae062

**Published:** 2024-06-07

**Authors:** Mohammad I Smerat, Balqis Shawer, Bara M AbuIrayyeh, Shahd T Natsheh, Laila Diab, Farah B Shahin, Mahmoud R Manasra

**Affiliations:** Radiology Department, Ahli Hospital, Palestine; College of Medicine and Health Sciences, Palestine Polytechnic University, Hebron, Palestine; College of Medicine and Health Sciences, Palestine Polytechnic University, Hebron, Palestine; College of Medicine and Health Sciences, Palestine Polytechnic University, Hebron, Palestine; College of Medicine and Health Sciences, Palestine Polytechnic University, Hebron, Palestine; College of Medicine and Health Sciences, Palestine Polytechnic University, Hebron, Palestine; College of Medicine and Health Sciences, Palestine Polytechnic University, Hebron, Palestine

**Keywords:** ceftriaxone, cholecystitis, cholecystolithiasis, drug-induced

## Abstract

Ceftriaxone is a third-generation cephalosporin. Due to its wide range of activity and acceptable safety profile, it is frequently prescribed to paediatric patients. However, there are several documented cases of reports of uncommon adverse events, such as cholecystitis, linked to the use of ceftriaxone. This study discusses the case of an 8-year-old female patient who developed cholecystitis, an inflammation of the gallbladder, after being treated with ceftriaxone. The patient presented with right upper quadrant pain, associated with nausea. Imaging studies showed the presence of stones and shadowing sludge, leading to acute inflammation of the gallbladder. Prompt cessation of ceftriaxone and supportive treatment led to the resolution of cholecystitis and the complete disappearance of the sludge and stones. The study highlights that early identification and withdrawal of the antibiotic can lead to successful therapy and the avoidance of unnecessary surgical procedures.

## Introduction

Ceftriaxone, a third-generation cephalosporin, is one of the most commonly used antibiotics for treating infections in paediatric patients [1]. Due to its extended spectrum of activity against gram positive and gram negative bacteria, long plasma half-life, low toxicity, and ability to penetrate cerebrospinal fluid with a high bactericidal titer, it is a good choice for treating bacterial meningitis [1].

Third-generation cephalosporins are typically well tolerated and have a low toxicity profile, as are the majority of beta-lactam antibiotics. A very uncommon side effect is gallbladder sludge and pseudolithiasis, which affect less than 0.1% of cases [2]. The literature shows improvement in this effect once the drug is discontinued [3].

In our case report, we present the case of an eight-year-old female patient with suspicious meningitis who developed acute cholecystitis with biliary pseudolithiasis after receiving ceftriaxone therapy.

## Case presentation

**Figure 1:**
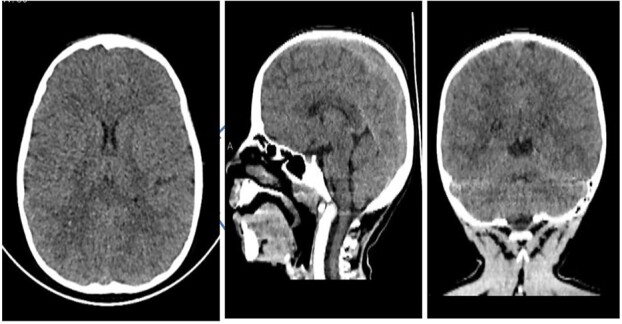
Axial, sagittal, and coronal views (from left to right) showing no abnormalities.

A previously healthy eight-year-old female patient presented to the emergency room with a headache, a high fever, and photophobia. Empirical treatment with ceftriaxone 2 g IV daily and acyclovir 400 mg orally three times daily was started immediately because of the high index of suspicion for bacterial meningitis. Non-contrast head computed tomography to rule out space-occupying lesions was done, which was normal (Fig. 1), and then a lumbar puncture was done. Cerebrospinal fluid analysis and culture results were consistent with the diagnosis. Her condition improved, and she was discharged on the seventh day.

Seven days later, the patient was brought to the emergency room by her family due to severe colicky right upper quadrant abdominal pain with radiation to the right flank of nine hours duration associated with nausea but no other associated symptoms. On physical examination, the patient looked ill and dehydrated. She was vitally stable, with a temperature of up to 37°C. On abdominal examination, right upper quadrant tenderness with a positive Murphy sign was noted. Her laboratory tests were as follows: normal hemoglobin levels (12.8 mg/dl), leukocytosis (WBCs 15.8 × 109/l), normal total bilirubin (0.2 mg/dl), normal direct bilirubin (0.1 mg/dl), normal aspartate aminotransferase (34 U/l), and normal alanine transaminase (14 U/l). The rest of her tests were normal. An abdominal ultrasound (US) was done, which showed a well-distended gallbladder containing a few stones with shadowing sludge (microlithiasis), a thickened wall, and a rim of pericholecystic oedema.

**Figure 2:**
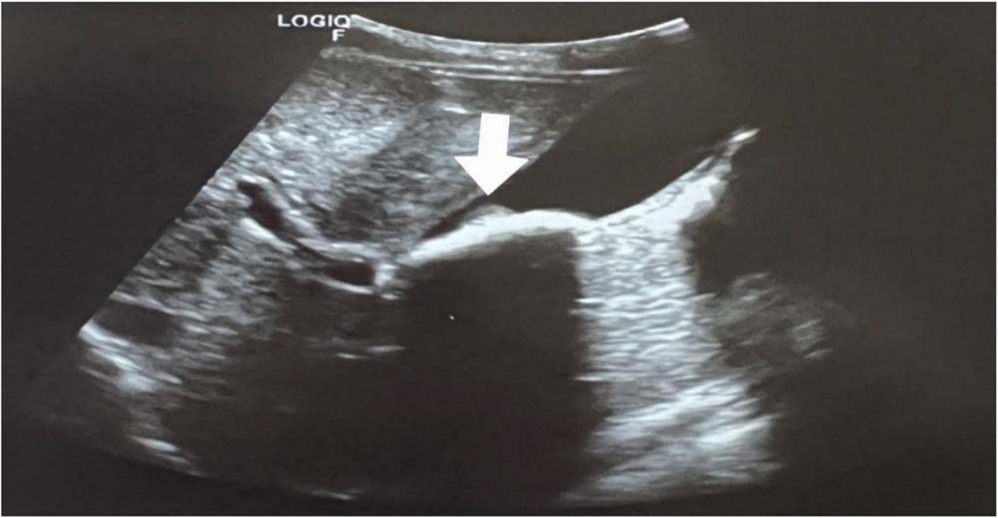
Axial view; ultrasonography of the gallbladder shows sludge with the stone formations (white arrow).

**Figure 3:**
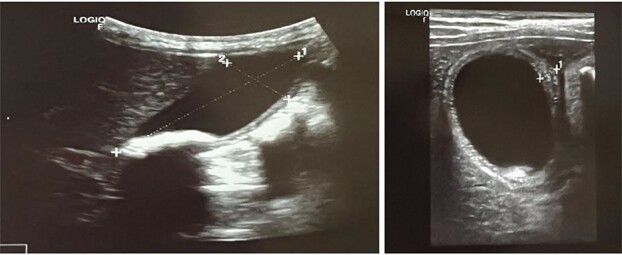
Axial and transverse ultrasonography view, sure wall thickness, and pericholecystic oedema.

The clinical and US findings were suggestive of acute calculous cholecystitis (Figs 2 and 3). This patient had no risk factors for gallbladder stone formation, such as liver diseases, hemolysis, or biliary tract infections, except for the ceftriaxone that was given to her on her first admission as a case of bacterial meningitis. According to this, she was diagnosed with ceftriaxone-induced acute cholecystitis.

The patient was admitted, kept Nothing by Mouth (NPO), and started on intravenous fluids, intravenous analgesics, and intravenous Cefuroxime 500 mg three times daily. Four days later, she was discharged.

**Figure 4:**
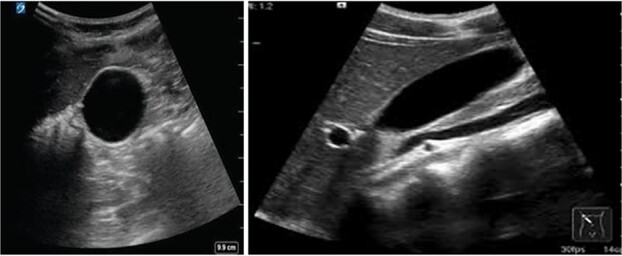
Normal gallbladder ultrasound after the drug discontinuation.

On follow-up visits, her laboratory tests were completely normal, and her ultrasound results showed a completely resolved inflammation with the complete disappearance of the gallbladder stones and sludge (Fig. 4).

## Discussion

Cholecystitis is an inflammation of the gall bladder that can occur acutely or in a chronic form, with or without the presence of a gallstone. Cholecystitis is a very unusual condition in the paediatric age group, with the incidence in children ranging from less than 1% to 4% in different studies [4].

Acute cholecystitis is accompanied by gallstones in 95% of adults, while the presence of gallstones in children with acute cholecystitis is less frequent compared to adults. When the cause of cholecystitis is the presence of stones, it seems that the process begins by blocking the duct with these stones, but how the inflammation process occurs is not clearly understood [5].

High doses or extended usage of ceftriaxone, dehydration, renal impairment, the geriatric population, and sepsis are risk factors linked to ceftriaxone-induced cholelithiasis [5]. With the exception of high-dose ceftriaxone, none of the risk factors listed above applied to our patient.

We conducted a literature review on reported cases of ceftriaxone-induced symptomatic cholelithiasis across PubMed between 1991 and 2022. To the best of our knowledge, only thirty cases have been reported in English-language literature. Sixteen of them are children; the male-to-female ratio was almost the same, with an overall mean age of 38. Cholelithiasis was discovered by ultrasound after a clinical complaint of acute cholecystitis. The majority of literature indicated that proloneed usage of ceftriaxone increased the incidence of cholelithiasis [6]. In our situation, cholelithiasis manifested after just seven days of ceftriaxone medication. Similarly, Biner et al. discovered that 27 out of 156 children (17%) had the onset of cholelithiasis within 3–7 days. [7]. Conservative treatment was the management of choice for the majority of patients, which included discontinuing the drug. The average cholelithiasis formation for all cases was after ten days, and follow-up evaluations were conducted after one month. In the majority of patients, cholelithiasis disappeared on US during follow-up [8, 9].

Due to their ultrasound-morphologic resemblance to gallstones and their quick disappearance after ceasing ceftriaxone therapy, they are referred to as ‘ceftriaxone pseudolithiasis.’

Pseudolithiasis and biliary sludge are typically asymptomatic and do not call for stopping treatment, according to studies. Despite this, reports of symptomatic instances of only a small percentage of ceftriaxone-treated patients developing symptoms and individuals who underwent cholecystectomy exist. However, it is advised to cease treatment when it becomes clinically evident [10]. In our case, follow-up with the patient four days after discharge showed completely resolved findings, both clinically and radiologically.

## Conclusion

We present an 8-year-old female patient whose condition developed after one week of intravenous ceftriaxone use. The diagnosis of ceftriaxone-associated biliary pseudocholelithiasis depends heavily on the clinical presentation, cholestasis markers, and abdominal imaging. Clinicians should be aware of this possibility to avoid unnecessary operations.

The limitation was a 3-point Naranjo Scale; points 1 to 4 give a definition of ‘possible.’ In addition, compared to the RUCAM system, the scale has lower sensitivity and specificity.
